# Discovery and mechanism studies of a novel ATG4B inhibitor Ebselen by drug repurposing and its anti-colorectal cancer effects in mice

**DOI:** 10.1186/s13578-022-00944-x

**Published:** 2022-12-21

**Authors:** Huazhong Xie, Pengfei Qiang, Yao Wang, Fan Xia, Peiqing Liu, Min Li

**Affiliations:** School of Pharmaceutical Sciences, Guangdong Provincial Key Laboratory of Chiral Molecule and Drug Discovery, National and Local United Engineering Lab of Druggability and New Drugs Evaluation, Sun Yat-Sen University, Guangzhou, 510006 Guangdong China

**Keywords:** ATG4B inhibitor, Autophagy, Colorectal cancer, FDA-approved drug, High- throughput screening, Protein oligomerization, Redox modification

## Abstract

Cysteine protease ATG4B, a key autophagy protein, is an attractive target for colorectal cancer therapy. However, ATG4B inhibitors with higher efficiency, safety, and clear mechanism are still limited. In this study, we discovered ATG4B inhibitors based on the FDA-approved drug library through FRET-based high-throughput screening and gel-based analysis. Among the nine hits, compound Ebselen showed the most potent ATG4B inhibitory activity (IC_50_ = 189 nM) and exhibited controllable selectivity and structural optimizable possibility against ATG4A and caspases. We then performed mass spectrometry assay and cysteine mutations to confirm that Ebselen could covalently bind to ATG4B at Cys74. Moreover, Cys292 and Cys361 instead of Cys74 are responsible for the redox-oligomerization and efficient activity inhibition of ATG4B. Ultimately through cell culture and mouse xenograft tumor models, we established the impact of Ebselen on autophagy and tumor suppression via ATG4B inhibition other than apoptosis. These results suggest that old drug Ebselen as an ATG4B inhibitor through oxidative modification may be repurposed as a promising anti-colorectal cancer drug.

**Supplementary Information:**

The online version contains supplementary material available at 10.1186/s13578-022-00944-x.

## Highlights

1. High-throughput screening of ATG4B inhibitors based on FDA-approved drug library
2. Ebselen can covalently bind to ATG4B at Cys74
3. Ebselen can promote ATG4B oligomerization at Cys292 and Cys361
4. Ebselen suppresses the growth of colorectal cancer cells and xenograft tumors via ATG4B inhibition

**Supplementary Information:**

The online version contains supplementary material available at 10.1186/s13578-022-00944-x.

## Introduction

Autophagy-lysosome system is a distinguished biodegradation pathway to maintain homeostasis commonly possessed in eukaryotes [1]. Appropriately, autophagy plays crucial roles in cellular physiology, including the clearance of waste products generated in response to metabolic stress, the turnover of developmental processes, and the protection against genomic damage, etc. It thus extends functions to prevent many diseases, including infections, cancer, neurodegeneration, and aging, among others [2].

Autophagy is fueled by types of biological machinery from *ATG* genes and requires two major ubiquitin-like systems: the ligases ATG7/ATG3/ATG5-ATG12 and ATG8/LC3-phosphatidylethanolamine (PE) conjugation systems. In brief, the Atg8 homologs of yeast, microtubule-associated protein 1 light chain 3 (MAP1LC3/LC3) or the GABA type A receptor-associated protein (GABARAP) subfamilies, are conservatively removed the extra amino acids at the C-terminus to expose the electrophilic glycine residue by catalytic enzymatic cleavage by the cysteine protease ATG4 family (ATG4A, ATG4B, ATG4C, and ATG4D). It then sequentially undergoes lipidated conjugation to the PE of the autophagosome membrane, besides, being stripped off by ATG4 in a similar manner [3, 4]. ATG4B is a key protease because of its ultra-efficient and nearly exclusive catalysis toward LC3B [5, 6]. Deletion or active site mutation of ATG4B all lead to the stalling of the autophagy pathway, interestingly also in the case of its high expression where an unexpected strong delipidation function [7, 8].

ATG4B has been implicated in several cancers, such as colorectal cancer (CRC) [9–11], gastric carcinoma [12], and breast Cancer [13]. Among them, ATG4B was validated to be highly expressed in CRC tumor tissues, and its knockdown resulted in blocking cell cycle progression and inhibition in CRC cells, typically HCT116 cell line [9]. To date, some published inhibitors of ATG4B had been tested in xenograft models of CRC cell lines and exhibited certainly tumor growth suppression. Recently, small molecule inhibitors have been increasingly reported with ATG4B as a target through TR-FRET/FRET-based [14–17], LC3B-PLA2 [11, 18], and gel-based assay [19, 20]. Among the well-known inhibitors are, covalent binding series based on substrate peptide-derived fluoromethylketone (FMK) [14], natural product-derived autrintricarboxylic acid [15], virtual screening derived LV320 [21], S130 [10], and another FDA-approved drug library derived Tioconazole [11]. So far, most of the discovered inhibitors had only been validated in vitro without relevant studies in vivo. The detailed inhibitory mode of those inhibitors for ATG4B has not been fully elucidated. Meanwhile, the few candidates that have been attempted in animal models had also not ushered in further development [10, 11], then safety and research costs may be the non-ignorable reasons for their stagnancy. Drug repurposing is a strategy to identify new uses for approved or investigational drugs. Its advantages such as adequate safety, shorter development time and investment cost [22], were paid our attention when screening of FDA-approved drug library.

In this study, a recognized FRET-based high-throughput screening assay was applied to obtain ATG4B inhibitors from an FDA-approved drug library. And after rigorous gel-based assay, selectivity and tentative structure relationship studies, we confirmed the potential of Ebselen to be an inhibitor for ATG4B. Ebselen was predicted and validated to be able to covalently bind and promote the oligomerization formation of ATG4B to highly inhibit its activity. Furthermore, Ebselen also suppressed autophagy and the growth of CRC cells and tumor xenografts. Taken together, Ebselen could be a promising and optimizable ATG4B inhibitor for related treatment of autophagy and cancers.

## Results

### Screening of ATG4B inhibitors based on the FDA-approved drugs library by FRET assay

**Fig. 1:**
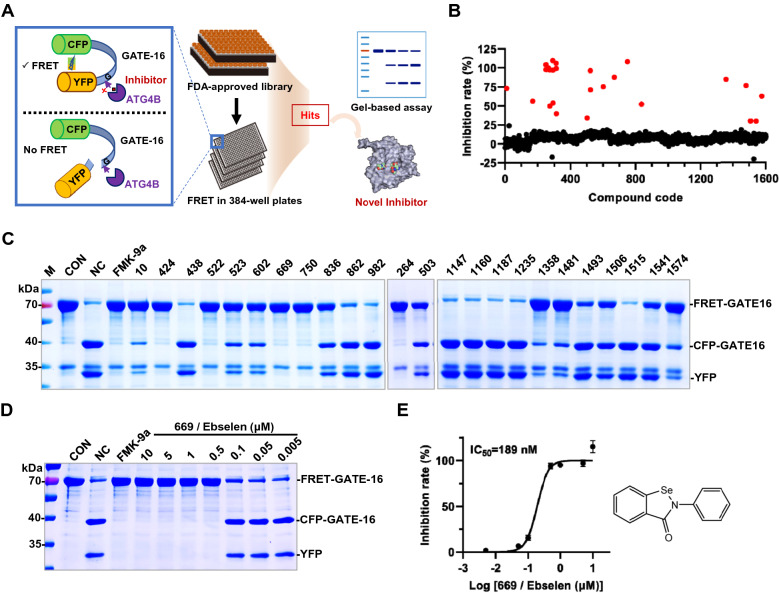
Screening of ATG4B inhibitors based on the approved drugs library by FRET assay. **A** Illustration of the FRET-based high-throughput screening platform for ATG4B inhibitors. **B** Scatter plot of inhibition rate of approved library for ATG4B based on FRET assay. Candidate compounds were marked in red. Recombinant ATG4B (0.75 μg/ml) was incubated with 1600 compounds (10 μM) from approved library for 30 min at 37 °C, then FRET substrates (0.75 μg/ml) were added and inhibition rates were determined as indicated. **C** The inhibitory effect of twenty-four candidate compounds (10 μM) were detected by SDS-PAGE and Coomassie brilliant blue (CBB) staining according to the cleavage of substrates. CON as control without enzymes, NC as negative control without inhibitor, FMK-9a as positive control. **D** The dose-dependent inhibition of compound **669** (Ebselen) were determined by SDS-PAGE. **E** IC_50_ calculation of compound **669** (Ebselen) for ATG4B from fitted curve by FRET assay and its structural formula

Taking ATG4B as the target, we used the high-throughput FRET-based assay to screen the FDA-approved drugs library containing 1600 compounds (Fig. 1A). After preparing the purified recombinant target protein ATG4B and the FRET substrate FRET-GATE-16 (CFP-GATE-16-YFP) (Additional file 1: Figure S1A, B), we tested the inhibitory effect of the reported positive compound S130 [10], which has a similar IC_50_ to that reported illustrating the successful establishment of the assay. (Additional file 1: Figure S1C). Then twenty-four candidate compounds (marked in red) were noted through an initial screening at 10 μM (Fig. 1B). To further validate their inhibitory effect, a more rigorous and authentic approach as gel-based assay was carried out (Fig. 1C). The brake of the cleavage reaction of the substrate FRET-GATE-16 was very intuitively reflective of the compounds’ inhibitory effect on the ATG4B enzymatic activity. Some fake positive compounds with weak inhibition such as compound **438** were excluded, and further nine commercially available compounds were subjected to IC_50_ determination (Additional file 1: Figure S1D). The IC_50_ values of most of the compounds showed close to 15 μM, meanwhile, compound **669** possessed the best inhibitory activity with an IC_50_ of 189 nM (Additional file 1: Figure S1D), compared with the value of 80 nM [14] for FMK-9a as well as 260 nM [23]. This was also verified in the FRET- and gel-based full concentration-response plot of compound **669** (Fig. 1D, E).

Totally, compound **669**, reported as Ebselen (SPI-1005, PZ-51, CAS 60940-34-3) was obtained from the FDA-approved library by high-throughput FRET assay.

### Ebselen is a highly active and selective inhibitor of ATG4B

**Fig. 2:**
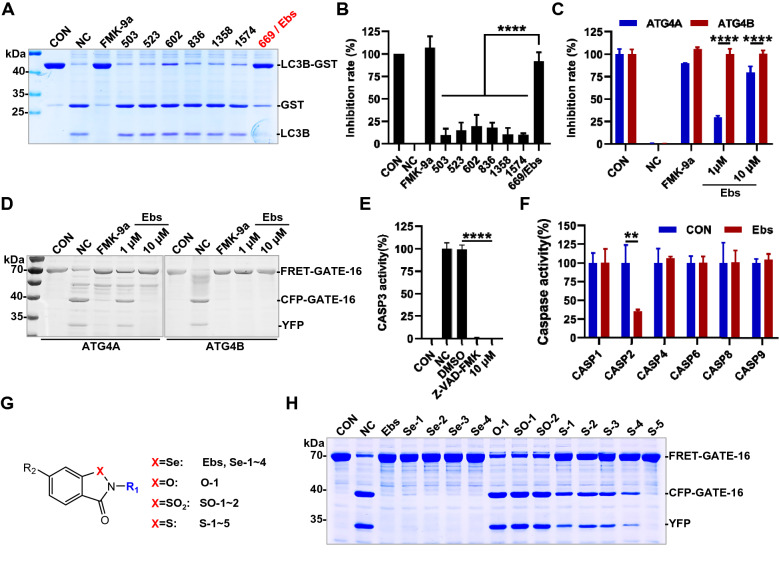
Ebselen is a highly active and selectivity inhibitor of ATG4B. **A**, **B** Cell lysates (1.5 μg) from ATG4B-Flag 293 T were incubated with different compounds (10 μM) for 30 min at 37 °C, then recombinant LC3B-GST (3 μg) were added up to 20 μl and inhibitions were detected by SDS-PAGE and CBB staining (**A**). **B** Statistical chart of gel results of three repeated experiments. Ebselen was abbreviated as Ebs, the same below. CON as control without lysate, NC as negative control without compound. **C**, **D** Inhibition rate of Ebselen for ATG4A and ATG4B determined by FRET assay (**C**) and validated by SDS-PAGE (**D**). CON as control without enzyme, NC as negative control without compound. **E** Target selectivity of Ebselen (10 μM) was tested against caspase-3 (CASP3). Z-VAD-FMK, 50 μM. CON as control without enzyme, NC as negative control without compound. **F** Target selectivity of Ebselen (10 μM) was tested against caspase proteases (CASP1, 2, 4, 6, 8, and 9). CON as control without Ebselen. **G** Structure list of analogues based on benzo[*d*] [1,2]selenazol-3-one scaffold. **H** The inhibitory effect of analogues (10 μM) was detected by SDS-PAGE and CBB staining

Inhibition potency for nine candidates was further estimated by in vitro cleavage assay of LC3B-glutathione-*S*-transferase (GST) with overexpression of ATG4B in cell lysates [5]. As shown in Fig. 2A, B, the cleavage activity of ATG4B could be still strongly suppressed by compound **669/**Ebselen, meanwhile other compounds showed poor activity in this assay.

The inhibition profile of endogenous ATG4B in cells was also noted, and as shown in Additional file 1: Figure S2A, B, ATG4B was inhibited in a time- and dose-dependent manner. Given that ATG4 possesses four isoforms (A, B, C and D), while ATG4C and ATG4D have little significant substrate enzymatic activity [6], so the selectivity of Ebselen for ATG4A and ATG4B was taken into account. In the FRET-based enzymatic cleavage experiments, it was obvious from Fig. 2C that the Ebselen had a weaker inhibition for ATG4A compared to ATG4B. Whereas in the validation results in gel (Fig. 2D), 1 μM of Ebselen gave cleaved substrate (CFP-GATE-16 and YFP) appearance compared to complete inhibition of ATG4B. In consideration of ATG4B as a cysteine protease, we determined the performance of the compound for some classical cysteine proteases, Caspase 1, 2, 3, 4, 6, 8 and 9 (CASP1, 2, 3, 4, 6, 8 and 9). As shown in Fig. 2E, F, Ebselen with the concentration of 10 μM had no significant effect on most caspase activities, but a certain inhibition on CASP2 and CASP3.

Further, structure-activity relationship (SAR) studies were implemented, based on the literature [24] and commercially available structural analogues. As is shown in Fig. 2G and Additional file 1: Figure S2C, the **Se** atom of the “benzo[*d*][1,2]selenazol-3-one” backbone remained unchanged, replaced with **S** atom, or **O** atom of similar chemical properties, as well as some simple side chain group changes. Among the compounds tested (Fig. 2H, Additional file 1: Figure S2C), the **Se** atom exhibited a striking inhibition (IC_50_ < 2 μM) and **S** atom being the second, while the **O** atom or the modified **S** atom (SO_2_, O=S=O) showed no inhibitory activity. Then the activity changes by side chain group were tentatively not regular, as these data came from irregular and few side chain alterations. Although we did not get lead compounds with better activity, it also suggested that structural modification was feasible. It is noteworthy that the inhibition of Ebselen on CASP3 had been explained, because pieces of literature had reported the clear inhibition of **S** atom analog Ebsulfur (CAS 2527-03-9) on CASP3, and related SAR studies and patents had been published [25, 26].

In general, Ebselen showed excellent inhibition effect in gel-based assay and exhibited controllable selectivity against ATG4A and caspases.

### Ebselen can covalently bind to ATG4B at Cys74

**Fig. 3:**
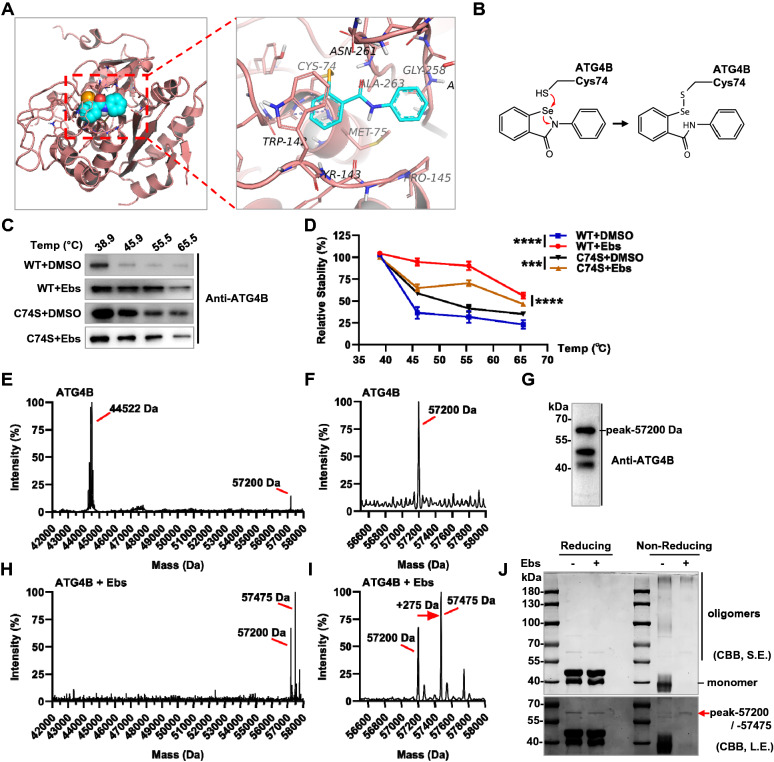
Ebselen can covalently bind to ATG4B. **A**, **B** Covalently bound molecular docking results and schematic sketch of ATG4B (PBD ID: 2Z0E) and Ebselen. Docking results plotted using PyMol. Pink as protein ATG4B, and blue as Ebselen. **C**, **D** Target engagements of Ebselen to recombinant ATG4B and C74S mutant protein in vitro thermal shift assay as indicated. **E**, **F** Mass spectrometry under denaturing condition of ATG4B only. **G** Sample from the mass spectrometry assay was subjected to western blot and validated with antibody against ATG4B. **H**, **I** Mass spectrometry under denaturing condition of ATG4B treated with Ebselen. **J** Samples from the mass spectrometry assay were subjected to reducing and non-reducing SDS-PAGE. S.E. as short exposure, L.E. as long exposure

The inhibitory mechanism and pattern of binding of the compound against ATG4B were next investigated. Firstly, a molecular docking study was performed, based on the current reported binding models of Ebselen [27]. We obtained that the compound was covalently bound to the Cys74 of ATG4B (Fig. 3A, B), which is precisely the mainly active catalysis site for ATG4B enzymatic cleavage. It was known that Trp142 was a key to switching the autoinhibitory-loop of ATG4B [28], and the compound just formed two pi-pi stacking interactions with Trp142, as well as other hydrophobic interactions with Tyr143, Pro145, Gly258, Ala263, etc. Subsequently, the ATG4B C74S mutant ATG4B^C74S^ (Cys74 to Ser74) was purified to perform in vitro thermal shift assay (TSA) for further studies (Additional file 1: Figure S3A) [29]. The thermal stability of the wild type (WT) ATG4B was strikingly improved compared with DMSO as control, while the stability of the C74S mutant significantly decreased (Fig. 3C, D). This revealed that the Ebselen may indeed covalently bind to ATG4B through the Cys74 site. Compared with the DMSO group, the stability of the C74S mutant was still a little increased with the compound (Fig. 3C, D), which also verified the existence of other interactions based on the docking results.

Mass spectrometry has always been able to provide direct evidence of protein-ligand binding [30]. Therefore, denaturing mass spectrometry was operated to prove the covalent binding of ATG4B with Ebselen. As shown in Fig. 3E, F and Additional file 1: Figure S3B, the spectrometry diagram of the protein sample without compound showed that there was a main peak of 44,522 Da (monomer molecular weight of ATG4B) and a small peak of 57,200 Da. SDS-PAGE and western blot were performed on the mass spectrometry samples, then the peak of 57,200 Da was indeed confirmed to be ATG4B (Fig. 3G). This form of ATG4B had also been observed in recent report [31], and it was speculated as an unknown aggregation form of ATG4B.

Then the compound Ebselen was added to the system, and surprisingly, the monomer ATG4B peak in the spectrum was disappeared (Fig. 3H). And the remaining two main peaks (57,200 Da and 57,475 Da) rightly had one compound molecular weight shift (Ebselen: *m/z* = 275 Da) (Fig. 3H–I), which showed that the Ebselen covalently binds to ATG4B with the 57,200 Da form. Non reducing SDS-PAGE is a good method to detect the aggregation form of protein, because the absence of reductant makes the disulfide bond remain. Interestingly, the monomer of ATG4B (~ 44 kDa) was also disappeared in non-reducing electrophoresis when treated with Ebselen, which was consistent with the results of mass spectrometry (Fig. 3H–J). Thus, we confirmed that Ebselen can covalently bind to ATG4B at Cys74 through in vitro TSA and mass spectrometry experiments.

### Ebselen can promote ATG4B oligomerization at Cys292 and Cys361

**Fig. 4:**
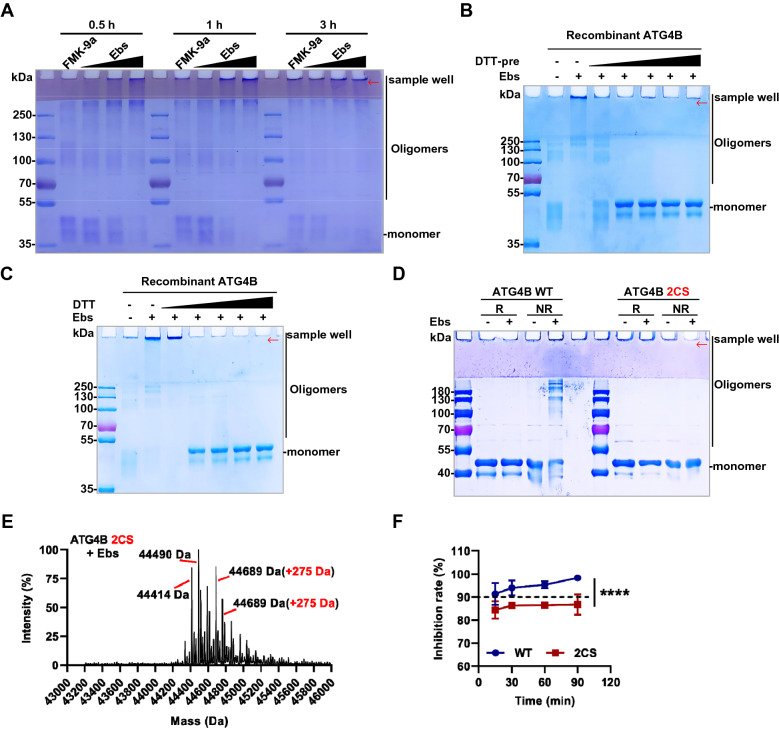
Ebselen can promote ATG4B oligomerization to highly inhibit ATG4B. **A** Recombinant ATG4B (5 μM) were incubated with FMK-9a (50 μM) and different concentrations (5, 25 and 50 μM) of Ebselen incubated for different time points (0.5, 1 and 3 h), and the results were detected by non-reducing electrophoresis. **B** Ebselen (50 μM) was incubated with different concentrations (0.05, 1, 2, 10, and 20 mM) of DTT for 30 min in advance, then recombinant ATG4B (5 μM) was added for another 30 min until submitted to non-reducing electrophoresis. **C** Recombinant ATG4B (5 μM) was incubated with Ebselen (50 μM) for 30 min before different concentrations (0.05, 1, 2, 10, and 20 mM) of DTT were added until subjected to non-reducing electrophoresis 30 min later. **D** Recombinant ATG4B WT and 2CS (C292/361S) mutant (5 μM) were treated with or without Ebselen (50 μM) and subjected to reducing or non-reducing electrophoresis. **E** Mass spectrometry under denaturing condition of ATG4B 2CS mutant treated with Ebselen. **F** Ebselen (0.5 μM) was incubated with ATG4B WT and 2CS mutant (0.75 μg/ml) for different time (15, 30, 60 and 90 min), and then the inhibition rates were detected by FRET assay

The disappearance of the ATG4B monomer made us have to consider a novel post-translational modification of ATG4B, namely, redox and oligomers formation [32]. Subsequently, ATG4B samples were incubated with different mole ratios (1:1, 1:5, and 1: 10) of Ebselen and for different time points (0.5, 1, and 3 h), and the results were detected by non-reducing electrophoresis. As shown in Fig. 4A, with the increase of the concentration of the compound or the extension of the incubation time, the ATG4B monomers were obviously gradually reduced, then oligomerization occurred, and even aggregates accumulated on the sample wells, finally. Notably, the classical covalent ATG4B inhibitor FMK-9a in the figure did not show similar properties, although a little aggregation occurred due to the prolonged oxygen exposure in the air (Fig. 4A). The aggregates formed could be reversibly regulated by a reducing agent, because an assay to add dithiothreitol (DTT) into the reaction was operated. Whether the compounds were incubated with different concentrations of DTT in advance (Fig. 4B), or the DTT was added after incubation between protein and compounds (Fig. 4C), ATG4B aggregates could return to the level of monomer in non-reducing electrophoresis. Furthermore, we also evaluated the redox properties of analogues on ATG4B. As is shown in Additional file 1: Figure S4A, most compounds with inhibitory effect can promote the oligomerization of ATG4B. This suggested that the ability to promote oligomerization may also be an evaluation aspect in the future structure optimization.

There were many studies on the redox of ATG4B, among which the most well-known reaction sites were Cys292 and Cys361, generating intermolecular disulfide bonds to form oligomers [32]. Therefore, we expressed and purified the mutant ATG4B^2CS^ (Cys292&Cys361 all to Ser) reportedly that could not undergo air-oxidation (Additional file 1: Figure S4B). It was intriguing that the 2CS mutant did not undergo oligomers modification when treated with Ebselen (Fig. 4D). These results clearly revealed that the oligomers of ATG4B formed by the compound depended on the classical site Cys292 and Cys361 rather than Cys74 (Fig. 4D and Additional file 1: Figure S4C). Naturally, we speculated that the covalent binding of the 2CS mutant with the Ebselen existed as the monomer and can be detected by mass spectrometry. And here in Fig. 4E, after two Cysteines were mutated to Serines (-32 Da), the molecular weight peaks of the 2CS mutant monomer were detected (44,490 Da, 44,414 Da, typically), and rightly covalent binding peaks with one compound molecular weight shift (275 Da) were also observed (44,765 Da, 44,689 Da). These above results demonstrated that Ebselen was able to covalently bind to ATG4B, especially the form of monomer. This also reconfirmed that the oligomerization of ATG4B after compound treatment was responsible for the disappearance of the monomer in the mass spectrometry.

Further, we verified the inhibition of ATG4B by oligomerization from Ebselen. As is shown in Fig. 4F, Ebselen showed higher inhibition rate against WT, compared to 2CS, which was less than 90%. Meanwhile, the WT group showed an increasing inhibition with increasing incubation time, while the 2CS showed almost no more increase. Interestingly, this result remained consistent with the increasing oligomerization of ATG4B in Fig. 4A. In other words, the gap in the inhibitory effect exhibited between WT and 2CS was a manifestation of the WT oligomerization modified by Ebselen.

In summary, ATG4B was also oligomerization modified due to oxidative regulation at Cys292 and Cys361, which might enhance the inhibitory effect of Ebselen on ATG4B.

### Ebselen suppresses autophagy flux via inhibition of ATG4B

**Fig. 5:**
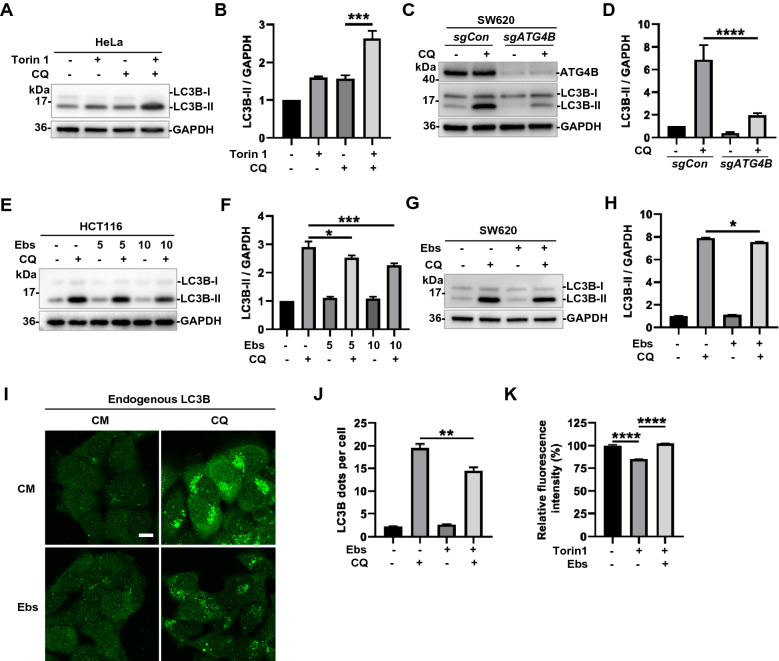
Ebselen suppresses autophagy flux via inhibition of ATG4B. **A**, **B** HeLa cells were treated with Torin 1 (2 μM) or CQ (40 μM) and detected by western blot. **C**, **D** SW620 cells were transduced with *sgCon* and *sgATG4B* lentiviruses, respectively. Then cells were treated with CQ (40 μM) and detected by western blot. **E**, **F** HCT116 were treated with Ebselen (5 and 10 μM) or CQ (40 μM) and subjected to western blot. **G**, **H** SW620 were treated with Ebselen (10 μM) or CQ (40 μM) and western blotting was carried out to detect the protein levels. **I**, **J** Immunofluorescence analysis of endogenous LC3B in HCT116 treated with Ebselen (10 μM) or CQ (40 μM). Dots numbers for LC3B were derived from counting at least 20 cells and 5 fields from three independent replicated experiments. Bar = 10 μm. **K** HCT116 cells were treated with 2 μM Torin 1 in the presence or absence of 10 μM Ebselen for 12 h, then the level ratio of long-lived proteins was measured by flow cytometry

Since ATG4B is a key protein in autophagy, the experiment on the effect of Ebselen on autophagy flux was worth proposing. First of all, we determined the most classic index LC3B for autophagy flux detection in HeLa cell line [33]. When the classic mTOR inhibitor Torin 1 and lysosomal inhibitor CQ (Chloroquine) were used together, the accumulation of LC3B-II indicated that Torin 1 was an autophagy inducer as commonly known and reported (Fig. 5A, B) [33].

Returning to the role of ATG4B on autophagic flux, when it was inhibited or knocked down, its effect on autophagy had been preliminarily concluded in some studies [9, 34, 35]. In our work, compared to the control, the addition of *sgATG4B* lentivirus in SW620 greatly reduced the protein level of ATG4B (Fig. 5C). Obviously, the level of LC3B-II was also reduced, greatly, when combined with CQ. It meant that knockdown of ATG4B brought about inhibition of autophagy flux (Fig. 5C, D). Such conclusion was also in line with most current studies. Since, in the latest theory, it was believed that ATG4B is involved in the growth and elongation phase of phagophore by interacting with ATG9A and dynamically regulating the lipidation/delipidation of LC3B [36, 37]. Similar results were obtained that the inhibition of autophagic flux was revealed by the reduction of LC3B-II, when the inhibitor Ebselen and CQ were used together in both HCT116 and SW620 cell lines (Fig. 5E–H). More intuitive and identical results were shown in Fig.  5I, J, the number of endogenous LC3B dots as autophagosomes was decreased when compound applied. Furthermore, a gold standard long-lived protein degradation assay was operated to confirm the inhibition. As is shown in Fig. 5K, Ebselen significantly inhibited autophagic long-lived protein degradation induced by Torin 1. In all, the inhibitory properties of Ebselen with respect to autophagy were comprehensively elucidated via inhibition of ATG4B.

### Ebselen suppresses the growth of colorectal cancer cells via inhibition of ATG4B

**Fig. 6:**
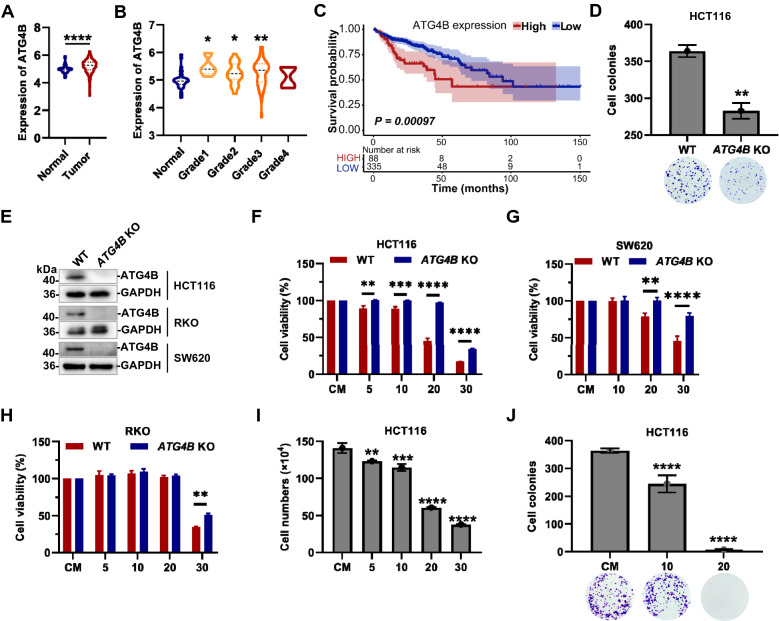
Ebselen suppresses the growth of CRC cells via ATG4B inhibition. **A** Bioinformatics analysis of ATG4B expression between normal (N = 41) and CRC tumor (N = 288) from the TCGA database. **B** Comparison of ATG4B expression between normal (N = 41) and different grades CRC tumor (grade1 N = 8, grade2 N = 40, grade3 N = 54, grade4 N = 4) from the TCGA database. **C** Survival analysis of ATG4B expression between high (N = 88) and low (N = 335) expression level from the TCGA database. **D** WT and *ATG4B *KO HCT116 cells were detected by colony formation assay. **E** The results of western blot validation of WT and *ATG4B *KO HCT116, RKO and SW620 cells. **F**–**H** WT and *ATG4B *KO HCT116, SW620 and RKO cells were treated with different concentrations of Ebselen and cell viabilities were detected by CCK-8 assay. (I) Cell numbers counting of HCT116 cells were treated with different concentrations of Ebselen. **J** HCT116 cells were treated with Ebselen and detected by colony formation assay

Reportedly, ATG4B was a potential therapeutic target for colorectal cancer (CRC, COAD), although not fully elucidated [9–11]. So further studies were urgently carried out, to demonstrate whether Ebselen could intervene or treat CRC models as a potential inhibitor of ATG4B. Firstly, the role of ATG4B in CRC was reaffirmed using bioinformatics analyses. The colorectal cancer data in the TCGA database were abstracted to investigate the relationship between COAD and ATG4B expression. As shown in Fig. 6A, the expression of ATG4B was significantly higher in CRC patients than in the normal subjects. Importantly, ATG4B expression in colon cancer cells (SW620, HCT116, and RKO) was indeed higher than that in normal colon cell (NCM460) (Additional file 1: Figure S5A). Moreover, in the occurrence and development in grades of colorectal cancer, high expression of ATG4B also appeared in grade 1, grade 2 and grade 3, which might be responsible for tumor deterioration (Fig. 6B). Meanwhile, by correlating ATG4B expression with survival curves, we concluded that high expression of ATG4B was a risk factor for CRC patients (Fig. 6C).

ATG4B knockout cells (HCT116 [10], RKO, SW620) were prepared, to confirm the effect on the growth of CRC cells when ATG4B was deficient (Fig. 6E). Firstly, colony formation assays were performed, and the ATG4B deficient cells formed significantly fewer colonies than the wild types (Fig. 6D). Cell viability was determined by CCK-8 assay, however, the effect of ATG4B deficiency or not on cell viability was not significant (data not shown). The difference became significant with the compound treatment. As shown in Fig. 6F-H, the growth of wild-type CRC cells (Particularly HCT116 and SW620) was significantly inhibited (typically, IC_50_ = 18.9 μM for WT HCT116), instead of the ATG4B knockout cell lines (IC_50_ = 28.1 μM for *ATG4B* KO HCT116). This also did reveal that the Ebselen was indeed working through ATG4B to suppress the growth of relevant colorectal cancer cell lines. The effects of the compound were also illustrated by cell counting and colony formation assays (Fig. 6I-J). And the results showed that Ebselen was able to significantly inhibit cell proliferation and colony formation.

Understandably, Ebselen showed nonselective cell killing at very high concentrations, so apoptosis detection was carried out. The appearance of cleaved poly ADP-ribose polymerase (PARP, substrate of the apoptosis execution protein CASP3) indicated the generation of apoptosis, when treated with the positive-induction compound Staurosporine (STS) (Additional file 1: Figure S5B-C). However, cells did not show any increase in apoptosis in response to treatment with low or high concentrations of Ebselen, in consist with the inhibition of CASP3 assayed earlier. In other words, these results above illustrated that Ebselen could indeed work to inhibit CRC cell proliferation via ATG4B, not via apoptosis. But the growth arrest effects of Ebselen on CRC cells may require further studies in the future, due to the dual inhibitory properties of the compound against both ATG4B and CASP3. However, it is noteworthy that recent studies suggested that inhibition of CASP3 could be also a potential target for cancer therapy. Because CASP3 and apoptosis could promote tumor growth, metastasis and angiogenesis in cancers, such as CRC, glioblastoma, gastric cancer, etc. [38–40].

In a word, we confirmed that Ebselen can suppress the growth of CRC cells via inhibition of ATG4B.

### Ebselen suppresses colorectal cancer xenograft tumor growth

**Fig. 7:**
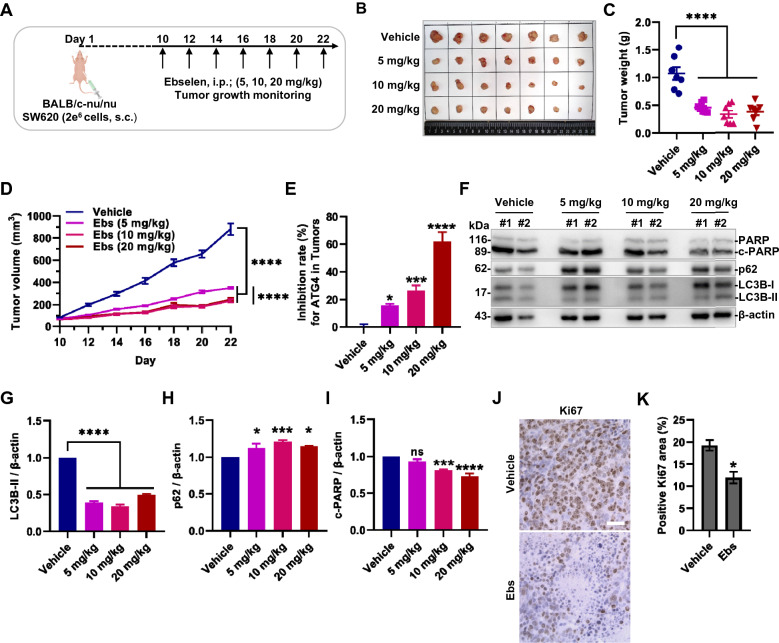
Ebselen suppresses colorectal cancer tumor growth. **A** A brief diagram of the animal experiment for subcutaneous xenografted tumor model. 2 × 10^6^ (2e^6^) SW620 cells were xenografted into BALB/c-nu/nu mice by subcutaneous injection (s.c.). After 10 days, mice were injected intraperitoneally (i.p.) with indicated doses of Ebselen every two days, and monitoring of tumor volume and body weight changes were performed. **B**–**D** Representative tumor images and tumor weight at the end time point were captured from SW620 cell-based xenografts. Mean tumor weight ± SEM (**C)** and mean tumor volume ± SEM (**D)** were shown. **E** The inhibitory effect of ATG4 in xenografted tumors was detected by FRET assay. **F**, **I** The protein level of autophagy and apoptosis in xenografted tumors were detected by western blot. Representative results were shown and the statistical results of each group were obtained from seven mice. **J**, **K** The immunohistochemistry analysis of Ki67 in xenografted tumors (group vehicle and Ebselen 10 mg/kg). Bar = 20 μm. The positive Ki67 area rate was obtained from the ratio of positive Ki67 area to total cell area by Image J

After the determination of the relevant in vitro cellular experiments, SW620 and HCT116 cell lines were xenografted into immune-deficient nude mice (BALB/c-nu/nu). Groups (Vehicle, 5 mg/kg, 10 mg/kg and 20 mg/kg, administration every two days) were generated randomly when tumors reached indicated sizes. The volume of tumors and body weight of mice were measured every two days (Fig. 7A). Compared with the vehicle group, the treatment group could significantly suppress the growth of tumors (Fig. 7B–D), and no obvious mice body weight change or organs damage was observed (Additional file 1: Figure S6A-B). Although the group of 5 mg/kg treatment had shown obvious anti-tumor effect, there was no difference between the medium and high concentration groups, which had better tumor suppression. Xenografts in BALB/c-nu/nu from HCT116 cell were also evaluated, showing a slightly weaker potential for tumor suppression (Additional file 1: Figure S6E–G).

After tumors were dissected, FRET assay and western blot were implemented to detect ATG4B activity and autophagy in tumors. As is shown in Fig. 7E, the activity of ATG4 (mainly ATG4B) in tumors was significantly inhibited in a dose-dependent manner. Then compared with the vehicle group, the autophagy flux in tumors of the administration groups was also significantly inhibited because of the reduction of LC3-II and the accumulation of the general autophagy cargo p62 (Fig. 7F–H, Additional file 1: Figure S6C-D). Meanwhile, Ebselen also inhibited the apoptosis of tumors in a dose-dependent manner (Fig. 7F, I). And we noticed that the background apoptosis in the vehicle group was higher, which was not coincident with the theory that the preceding apoptosis promoted the occurrence and development of tumors in some ways. So apart from the inhibition of ATG4B to generate antitumor effect, on the other hand, the inhibition of CASP3 also cannot be simply ignored. Overall, the results obtained from tumors were in keeping with the results of the previous in vitro and cellular experiments.

The tumor therapeutic potential of Ebselen via ATG4B was also further evaluated by immunohistochemical staining for Ki67, an indicator of tumor malignant proliferation (Fig. 7J, K). It showed a significant reduction of Ki67 positivity in the drug-treated group, demonstrating some reduction in tumor proliferation and malignancy. Taken together, Ebselen can suppress colorectal cancer xenograft tumor growth in vivo.

## Discussion

Autophagy plays an important role in the occurrence, development, deterioration and metastasis of CRC. And accumulation of the autophagic marker LC3B on autophagosomes had been seen in advanced case reports of CRC patients [41, 42]. In other words, to treat CRC and improve the chances of recovery, it may be necessary to reduce LC3 or inhibit autophagy. A dual-acting ‘switch’ in the autophagy signaling pathway is the cysteine protease ATG4, which both primes the pro-LC3 and recycles the lipidated form LC3-II/PE, thus bringing them back to LC3-I, prompting the orderly progression of autophagy [6, 7]. Among the four isozymes, ATG4B had the best protease activity and has been demonstrated to promote tumorigenesis and malignancy [9], thus it emerged as the most potential intervention and therapeutic target. In this study, we employed a classical drug screening platform that integrates a high-throughput FRET-based and reliable gel-based assay, to screen an FDA-approved drugs library. Then we report as follows, firstly, that drug-repositioning of Ebselen indicated the ability to covalently bind at Cys74 and efficiently inhibit the enzymatic activity of ATG4B. Second, Ebselen promoted redox oligomerization modification of ATG4B and relied on canonical Cys292 and Cys361 sites, with multiple reversible mechanisms of activity regulation. Finally, the compound also inhibited autophagic flux and suppressed CRC tumor model growth. These findings suggested that Ebselen was a potential drug to anti-CRC based on the target of ATG4B. In addition, Ebselen was an FDA-approved drug, indicating that its preliminary safety was warranted and more highly likely to have new indication in CRC therapy.

Significantly, the limitations of reported inhibitors had been repeatedly mentioned [10, 34], and there was currently no unified inhibitor backbone for ATG4B. Therefore, inhibitors with high bioactivity and safety profiles with new scaffolds deserve further development. In our work, an FDA-approved drugs screening was applied, employing the most established FRET assay and the most reliable gel-based assay. Compared with the current reported compounds with high inhibitory activity, the in vitro inhibitory effect of Ebselen was close to them with IC_50_ of 189 nM. Meanwhile, Ebselen also maintained a high inhibition effect in cell lysate assay, and also had a good selectivity between ATG4B and ATG4A. However, among the numerous Caspase proteins, the compound exhibited its dual target inhibitory properties with visualized inhibition for CASP3. This was understandable based on its relatively small compound backbone, and SAR studies had been performed with Ebsulfur for CASP3, a structurally similar sulfur compound. But preliminary “benzo[*d*][1,2] selenazol-3-one” backbone-based SAR studies gave us confidence in the future structure optimization, because the changes in the activity of the backbone were conservative. And two structural modification routes were pointed out, one was to improve the selectivity and higher bioactivity of the compounds according to the binding pocket characteristics of ATG4B. On the other hand, structural optimization was to preserve the scaffold’s natural dual target inhibitory function, but more disease applicability of the dual targets required to be investigated.

The structural coordinates of ATG4B had been deposited in the PDB database at present [28, 43], but no co-crystal complex containing inhibitors. The pattern of inhibition of ATG4B by inhibitors was therefore poorly studied. Interestingly, in this work, we gave an exhaustive insight into why and how Ebselen inhibited ATG4B. First, because of the reported high reactivity of the bond between the Selenium and the Nitrogen atom in the scaffold [27, 44], it was natural for Ebselen to covalently bind to ATG4B as predicted by molecular docking. Subsequently, in vitro thermal shift assay and the most accepted denaturing high-resolution mass spectrometry assay indeed confirmed this relationship. Unlike the currently famous covalent inhibitor FMK-9a [14, 23], Ebselen possessed different covalent reaction principles and more optimal structural remodeling space. Interestingly, we observed the disappearance of monomer ATG4B in the case of compound treatment during the mass spectrometry experiment, which was reminiscent of another widely studied property of ATG4B. Redox regulation was an important property of cysteine protease ATG4B, and many current studies had shown that ATG4B activity is regulated by ROS and redox [32, 45, 46]. We have reported that sites Cys292 and Cys361 of ATG4B could form intermolecular disulfide bonds as a result of oxidation, thereby converting the monomeric form to an oligomerized form [32]. Likewise, when treated with Ebselen, ATG4B abolished the monomeric form and converted to oligomers and even aggregates, which could not be monitored by mass spectrometry. Further, the role of 2CS sites in this reaction perfectly verified that oligomerization resulting from Ebselen treatment did not occur. Notably, however, Ebselen had evolved more among the reported properties to neutralize ROS, that is, some reducibility [47, 48]. In another redox reaction [49], Ebselen was as capable of being reduced to selenenyl sulfide intermediate by the strong reducing agent glutathione (GSH), so it was not difficult to understand that Ebselen could take away the proton on the reactive Cysteine residues and promote the formation of intermolecular disulfide bonds (Additional file 1: Figure S4D).

The role of knockdown of ATG4B in autophagic flux was currently slightly controversial, as there were reports that knockdown showed effects of inducing and inhibiting autophagy, respectively [9, 35]. However, in the autophagy flux studies of related inhibitors, it almost exclusively showed the inhibition of autophagy flux. Interestingly, changes of classic markers LC3B and p62 of autophagy had been mixed. On one side, autophagy under treatment with the small molecule compounds Tioconazole and S130 manifested as accumulation of the lipidated form LC3-PE/-II, which may result from the inhibition of ATG4B delipidation by the compounds [10, 11]. On the other hand, puncta of LC3 or autophagosomes formation were shown to be blockaded, in the case of classical NSC185058 and the latest long-chain compound **17** treatments [20, 34]. Similar to the latter, Ebselen in this study was able to block the formation of LC3-II or accumulation of autophagosomes in already arrested autophagy by CQ. Taken together with the present studies and perspectives, ATG4B also played an important role in the formation of the phagophore and the maturation of the autophagosome, including interacting with ATG9A and dynamically regulating LC3 up-and-down to membranes to promote autophagosome growth [36, 37]. Thus, the way to measure the effects of ATG4B inhibition would become more comprehensive and complex as studies progress.

Autophagy promoted tumorigenesis and malignant development, such as up-regulated cancer stem cell survival, resistance to nutrient-poor environments, and restricting the chemosensitivity and radiosensitivity of CRC [42, 50]. Thus, we could not deny that Ebselen played some role in suppressing the tumor model malignancy by inhibiting autophagy via ATG4B. But based on the current studies [9], the potential of ATG4B to generate CRC-obstructing effects independent of autophagy was still not fully unleashed. Interestingly, Ebselen was able to achieve ~ 50% inhibition of the viability of CRC cell lines while showing almost no effect on ATG4B deficient cells (Fig. 6F). In addition, dual targets inhibition might both work in tumor model suppression, because increasing numbers of studies supported that CASP3 could be also a target for tumor therapy [38, 51, 52], which needed further clinical analyses. Regarding previous studies on Ebselen [47, 48], which also has anti-inflammatory, antioxidant effects, etc., then we believe that these potential effects may be beneficial in CRC therapy. Moreover, the latest debuts of Ebselen were on the potential therapeutic for COVID-19 and neuroprotective effect [27, 53]. Although these above roles, need to be further investigated, are targeted at different targets, such as M^pro^ for SARS-CoV-2 [27], IMPase for bipolar disorder [53], and Ag85C for infectious disease [44], etc. Although Ebselen had been included in several clinical trials before, so far, no specific prospect of clinical application and drug use have been seen. In addition, Ebselen was best known among selenium organic compounds. And although it was nonspecific due to its small scaffold, it had proven to be always well tolerated in vivo, exhibiting no toxicity, and deserving richer bioactivity studies.

In conclusion, we have identified an FDA-approved ATG4B inhibitor based on its high potency in ATG4B inhibition both in vitro and in vivo, and generating desirable suppression of autophagy and proliferation of xenografted CRC cell tumors. In addition, the activity and function of ATG4B were regulated in several ways, and the development of its inhibitors may also become a promising direction for cancer therapy. Based on our data shown in this study, the bioactivities of Ebselen in this study and previous related studies seem to confirm it be eligible for further related studies or improvements. These findings provide a crucial role of Ebselen in anti-cancer growth and underscore the potential of targeting ATG4B as a novel strategy for colorectal cancer therapy.

## Materials and Methods

### Reagents and antibodies

The FDA-approved Drug Screening Library (L1000) was from TargetMol (Boston, MA). Candidate compound **10** (M832611), **424** (B802495), **503** (H811537), **523** (L830883), **602** (O815322), **836** (R830529), **1574** (S832949) were purchased from Macklin (Shanghai, China) and compound **669**/Ebselen (E3520) and **1358** (232,120) were from Sigma (St. Louis, MO). S130 [10] and FMK-9a [23] were preserved in our lab. Z-VAD-FMK (S7023) and Staurosporine (STS, S1421) were from Selleckchem (Houston, TX). Chloroquine (CQ, A506569) was from Sangon Biotech (Shanghai, China). The sources of different atomic analogues of Ebselen were as follows: **Se** atom analogues of Se-1 ~ 4 were a gift from professor Kewu Yang (Northwest University), and other **O**, **SO**, **S** atom analogues were from BidePharm (Shanghai, China).

The antibody ATG4B (15,131-1-AP) and GAPDH (60,004-1-Ig) was from Proteintech (Wuhan, China), LC3B (L7543) was purchased from Sigma (St. Louis, MO), antibody anti-LC3B (PM036) for immunofluorescence was from MBL (Beijing, China), Secondary antibodies conjugated with peroxidase and Alex Fluor-488 were from ThermoFisher Scientific (Waltham, MA).

### Protein expression and purification

The purification of Recombinant 6xHis-tagged protein ATG4A, ATG4B, ATG4B^C74S^ mutant, ATG4B^C292/361S^ mutant and FRET substrate FRET-GATE-16 (CFP-GATE-16-YFP) were described before [17, 32]. In brief, the BL21(DE3) *Escherichia coli* cells (Weidi Biotechnology, EC1002) transformed with the related plasmids were grown in LB medium at 37 °C until the OD_600_ reached 0.8. Then 0.5 mM isopropyl β-D-thiogalactopyranoside (IPTG, Sigma, I6758) was added to induce overnight at 16 °C. The cells were harvested and resuspended in lysis buffer (500 mM NaCl, 20 mM Tris pH 8.0, 20 mM imidazole, 2 mM 2-mercaptoethanol). After ultrasonic and filtration, the supernatants were loaded onto Ni–NTA agarose (QIAGEN, 30210). After washing with lysis buffer, the target proteins were eluted with buffer (500 mM NaCl, 20 mM Tris pH 8.0, 200 mM imidazole, 2 mM 2-mercaptoethanol) and buffer-exchanged into storage buffer (150 mM NaCl, 20 mM Tris–HCl, pH 8.0, 1 mM DTT) before stored at −80 °C.

### FRET-based assay and Caspase proteases activity measurement

FRET-based assay for ATG4B or ATG4A activity was operated as previously described [5, 17]. Briefly, Recombinant protein ATG4B (0.75 μg/ml) or ATG4A (5 μg/ml) was incubated with compounds in reaction buffer (150 mM NaCl, 20 mM Tris–HCl, pH 8.0, 0.1% CHAPS, 1 mM EDTA) at 37 °C. After 30 min, substrate FRET-GATE-16 (50 μg/ml) was added to a final volume of 50 μl. The reaction was recorded via relative fluorescence intensity ratio of 527 nm/477 nm (RFU). The final inhibition rate of the compound for ATG4 was calculated as: (RFU_compound_-RFU_NC_)/(RFU_CON_-RFU_NC_) × 100%; respectively, NC as negative control without inhibitor, CON as control without enzymes. For ATG4 activity determination in cells and tissues, cells or separated tumor samples were homogenized in a native lysis buffer (150 mM NaCl, 20 mM Tris–HCl, pH 7.6, 1% TritonX-100, 1 mM EDTA) without protease inhibitors. Then 5 μg total proteins from each sample was added into FRET assay system after clean supernatant proteins were separated. The reaction was recorded via relative fluorescence intensity ratio of 527 nm/477 nm. Finally, the inhibition rates of ATG4B were calculated with the vehicle or CM group as the negative control (ATG4 activity defined as 100%).

The enzyme activity of Caspase 1, Caspase 2, Caspase 3, Caspase 4, Caspase 6, Caspase 8 and Caspase 9 were determined via the activity assay kits (C1101, C1107, C1115, C1121, C1135, C1151 and C1157) from Beyotime (Shanghai, China). Then the experimental procedures were performed according to the manufacturer’s protocols.

### Molecular docking study and in vitro thermal shift assay

The study was performed based on the crystal structure of ATG4B (PDB code: 2Z0E, http://www.pdb.org). For molecular docking calculations, the files for the protein and ligand were prepared according to the protocol from Maestro (Schrödinger, LLC, NY). All docking parameters were conserved to default values of covalent docking, except Cys74 of ATG4B assigned as the active site.

For in vitro thermal shift assay, recombinant protein ATG4B or C74S mutant (100 nM) was incubated with Ebselen (1 μM) (DMSO as control) in the PCR tubes. The PCR instrument program was set at 25 °C for 10 min, 37 °C to 70 °C for 30 min and followed by cooling tubes for 5 min at room temperature. Then centrifuged at 12,000 rpm for 10 min at 4 °C, soluble fractions were separated from precipitates to be analyzed by SDS-PAGE and immunoblotting.

### Cell culture and preparation of ATG4B knockout cell lines

HeLa, HCT116, *ATG4B* KO HCT116 [10], RKO, *ATG4B* KO RKO, ATG4B-Flag 293 T [32] were cultured in Dulbecco’s modified Eagle’s medium (Gibco, 12,800,082) supplemented with 10% (v/v) fetal bovine serum (Sigma, 12,103) and 1% penicillin/streptomycin at 37℃ in 5% CO_2_ incubator (ThermoFisher). SW620 and *ATG4B* KO SW620 were cultured in RPMI 1640 medium (Gibco, 61,870,036). For the preparation of ATG4B knockout RKO and SW620, the verified lentivirus particles [10] were added to the indicated cells for 24 h, supplemented with 8 μg/ml of polybrene (YEASEN, 40804ES76). Subsequently, after puromycin resistance screening, the cells were amplified and sorted into single clones until the ATG4B-deficient cell lines identified by western blot.

### Immunoblotting and immunofluorescence staining

Immunoblotting assay was performed reportedly previously [45]. Commonly, cells were lysed in ice-cold RIPA lysis buffer (Beyotime, P0013B) supplemented with Pierce™ protease inhibitor tablet (ThermoFisher Scientific, A32965). Then typically 20–30 μg clean total proteins were loaded into 12% SDS-PAGE gel and followed by transfer to the PVDF membranes (Merck Millipore), before blocking with 5% non-fat milk in TBST buffer (137 mM NaCl, 20 mM Tris–HCl pH 7.6, 0.1% Tween 20) for 1 h. The membranes were washed with TBST three times before being incubated with primary antibodies overnight at 4℃. Then the membranes were incubated with secondary antibodies to be visualized by Image analyzer (Tanon, 5200).

Immunofluorescence staining was carried out as described before [45]. Respectively, cells seeded on glass-bottom 35-mm dishes (SORFA bio, 201,200) were washed with PBS and fixed for 20 min at room temperature with 4% paraformaldehyde after related administration. Subsequently, dishes were permeabilized with 0.3% Triton X-100 in PBS, and washed to be blocked in goat serum (Boster, AR1009) for 1 h before incubated with primary antibody overnight at 4℃. Confocal images were acquired with FV3000 (Olympus) supplemented with Alex Fluor-488 labeled secondary antibody.

### Mass spectrometry

Analysis of covalent binding between protein and ligand was performed by denaturing mass spectrometry. ATG4B WT and related mutant (20 μM) were incubated with or without compound (60 μM) in phosphate buffer for 1 h at 37℃. Then the samples were desalted using ultrafiltration spin columns (0.5 ml, 10 kDa MWCO, PES, Sartorius) and buffer-exchanged into Milli-Q water. Subsequently, samples were diluted containing 0.1% v/v formic acid before being subjected to denaturing electrospray ionization mass spectrometry analysis (TOF, Waters, SYNAPT G2-Si). Spectrum data were processed with MassLynx (Waters).

### Long-lived protein degradation assay

Long-lived protein degradation assay was performed referring to the reported protocol [54]. In brief, HCT116 cells were seeded in 6-well plates and cultured overnight, and then incubated with L-methionine-free DMEM (Thermo Fisher Scientific, 21,013,024) supplemented with 10% dialyzed FBS (Biological Industries, 2,148,391) for 30 min at 37℃ for depleting the methionine reserve. Subsequently, the cells were incubated with medium containing 50 μM AHA (MCE, HY-140346A) for 18 h to label AHA on proteins. After 2 h incubation with 10 × L-methionine to chase out the short-lived AHA-labeled proteins, cells were treated with compounds as indicated in regular medium for 12 h. Then the cells were harvested, fixed and permeabilized sequentially until they were incubated in the click reaction master mix (50 μM TAMRA alkyne [AAT Bioquest, AAT-487], 1 mM TCEP [MCE, HY-W011500], 100 μM TBTA [GLPBIO, GC45003], 1 mM CuSO_4_ [Sangon Biotech, C3008] in PBS) in the dark for 2 h. Finally, cells were washed and suspended in 500 μl PBS and analyzed by flow cytometry (BECKMAN, CytoFLEX S).

### Bioinformatic analysis of ATG4B in COAD

The bioinformatic analysis was operated with Sangerbox3.0 (www.sangerbox.com). In brief, the unified standardized pan-cancer dataset TCGA was downloaded from UCSC (https://xenabrowser.net/). Then expression data of ATG4B (ENSG00000168397) gene in each sample were extracted and filtered out the expression level of zero. Subsequently, log2(x + 1) transformation of each expression value were performed, before the expression differences between normal and tumor samples were calculated and plotted. We used the data of 423 COAD patients who completed clinical annotation for survival analysis of ATG4B, and determined the optimal cut-off point using the survmineR package, divided the patients into high and low ATG4B expression groups, before Kaplan Meier curves plotted.

### Colony formation and cell growth determination

For colony formation, WT HCT116 and *ATG4B* KO HCT116 cells were seeded in 6-well plates at 800 cells per well and cultured for 9–12 days, respectively. Concurrently, medium containing indicated compounds were replaced every 3 days, before the cells were fixed in 4% paraformaldehyde for 20 min and washed with PBS three times. Then colonies were stained with crystal violet (Beyotime, C0121) for 1 h and excess dyes were washed with PBS. Eventually, colonies more than 1 mm were counted in at least three independent experiments.

WT/*ATG4B* KO HCT116, WT/*ATG4B* KO SW620 and WT/*ATG4B* KO RKO cells were seeded in 96-well plate at 1000–1500 cells per well for cell viability assay, and seed in 6-well plate at 1 × 10^5^ cells per well for cell number counting. After 12 h, the indicated drugs were added to the medium and cultured for another 96 h. Total viable cell numbers were counted by a cell counter (BioRAD), and cell viability was determined by cell-count-8 assay kit (CCK-8, Bimake, B34304) according to the manufacturer’s protocol.

### Tumor xenograft studies

Four- to six-weeks-old BALB/C nu/nu female mice purchased from the Experimental Animal Center of Sun Yat-Sen University, were approved by the Animal Ethics Committee at Sun Yat-sen University and kept in specific-pathogen-free environment. Approximately 2 × 10^6^ SW620 cells or 1 × 10^7^ HCT116 cells were injected subcutaneously into the dorsal flank on the right side of the mice. Different groups were divided randomly when the tumor volume was approximately 60–80 mm^3^, before drug administration intraperitoneally (i.p.). Tumor volume was measured by calipers and calculated using the equation: 0.5 × length × width^2^. At the end of the experiments, the mice were sacrificed and tumors were dissected and weighed. In particular, we employed Servicebio (Wuhan, China) to carry out H&E staining and immunohistochemistry assay.

### Statistical analysis

All results were expressed as means ± SEM/SD of at least three independent experiments, and plotted using Prism 6.0 (GraphPad, La Jolla, CA). Statistical analyses were performed using paired/unpaired Student’s t-Test or one-way ANOVA. And values of *P < 0.05 were considered as being significant.

## Supplementary Information

**Additional file 1: Figure S1.** Screening of ATG4B inhibitors based on the approved drugs library by FRET assay. (A-C) FRET-based assay was established with recombinant proteins ATG4B (A) and FRET-GATE-16 (B), and validated with IC_50_ determination of positive compound S130 (C). (D) Candidate compound number and name correspondence table, and IC_50_ calculation of nine commercially available compounds for ATG4B by FRET assay. **Figure S2.** Ebselen is a highly active and selectivity inhibitor of ATG4B. (A-B) The dose- and time-dependent inhibition of Ebselen in HeLa cells were determined by FRET assay. CON as control without cell lysate, CM as complete medium treated group. (C) Specific structure list of analogues based on benzo[*d*][1,2]selenazol-3-one scaffold. **Figure S3.** Ebselen can covalently bind to ATG4B. (A) The purified recombinant ATG4B C74S mutant was subjected to SDS-PAGE. (B) Detailed characteristic peaks of ATG4B in denaturing mass spectrometry. **Figure S4.** Ebselen can promote ATG4B oligomerization to highly inhibit ATG4B. (A) Recombinant ATG4B (5 μM) was incubated with Ebselen-like analogues (50 μM) for 30 min and detected by non-reducing electrophoresis. (B) The purified recombinant ATG4B 2CS mutant was subjected to SDS-PAGE. (C) Recombinant ATG4B C74S mutant (5 μM) were treated with or without Ebselen (50 μM) and subjected to reducing or non-reducing electrophoresis. (D) A sketch of the redox properties of Ebselen. Ebselen cloud covalently bind to ATG4B Cys74 and induce oligomerization modification of ATG4B, which above regulated by the reducing agent DTT. **[O]** represents reactive oxygen species. Mono as monomer and Oligoes as oligomers. **Figure S5.** Ebselen suppresses the growth of CRC cells via ATG4B inhibition. (A) Western blot results of ATG4B expression in colonic epithelial cell line NCM460 and colon cancer cells. (B) WT and *ATG4B KO* HCT116 were treated with Staurosporine (STS, 2 μM) or Ebselen for 6 h and detected by western blot. (C) WT and *ATG4B KO* HCT116 cells were treated with different concentrations of STS and cell viability were detected by CCK-8 assay. **Figure S6.** Ebselen suppresses colorectal cancer tumor growth. (A) The body weight change rate of the mice in all groups showed no obvious change. (B) Representative H&E staining images were captured from mice liver and kidney tissues (group vehicle and Ebselen 10 mg/kg). Bar = 20 μm. (C-D) The protein level of autophagy and apoptosis in xenografted tumors were detected by western blot. (E–G) Animal experiment of HCT116 based xenograft in BALB/c-nu/nu mice. Mean tumor volume ± SEM (F), mean tumor weight ± SEM (G) and body weight change rate (H) were shown.

## Data Availability

Not applicable.

## Abbreviations

ATG: Autophagy-related
CASP3: Caspase-3
CQ: Chloroquine
CRC: Colorectal cancer
DTT: Dithiothreitol
Ebs: Ebselen
FRET: Förster resonance energy transfer
LC3B: Microtubule-associated protein 1 light chain 3 beta
SAR: Structure-activity relationship
TSA: Thermal shift assay
