# Supplementary figures and images for: Discovery and mechanism studies of a novel ATG4B inhibitor Ebselen by drug repurposing and its anti-colorectal cancer effects in mice
Source: Cell Biosci. 2022 Dec 21;12:206. doi: 10.1186/s13578-022-00944-x (PMC9767854; doi:10.1186/s13578-022-00944-x)

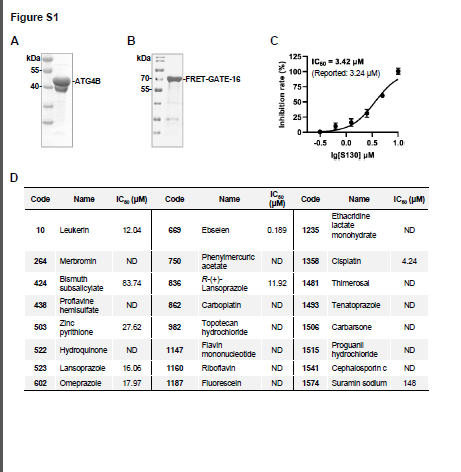


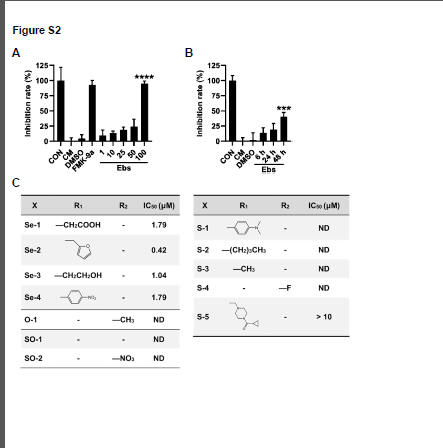


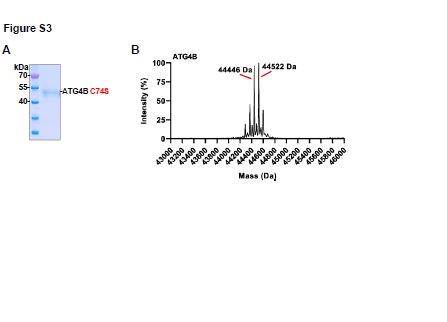


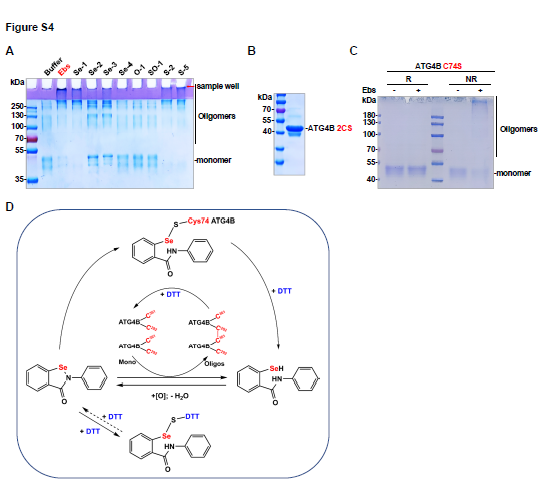


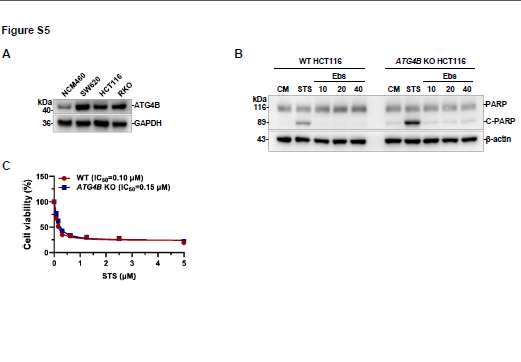


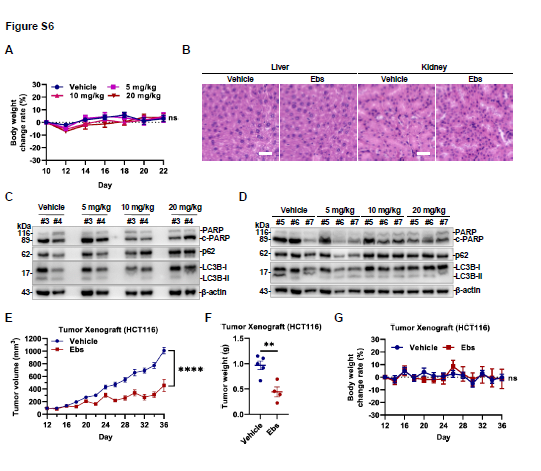

Supplement: Supplementary file 1 — Additional file 1: Figure S1. Screening of ATG4B inhibitors based on the approved drugs library by FRET assay. (A-C) FRET-based assay was established with recombinant proteins ATG4B (A) and FRET-GATE-16 (B), and validated with IC50 determination of positive compound S130 (C). (D) Candidate compound number and name correspondence table, and IC50 calculation of nine commercially available compounds for ATG4B by FRET assay. Figure S2. Ebselen is a highly active and selectivity inhibitor of ATG4B. (A-B) The dose- and time-dependent inhibition of Ebselen in HeLa cells were determined by FRET assay. CON as control without cell lysate, CM as complete medium treated group. (C) Specific structure list of analogues based on benzo[d][1,2]selenazol-3-one scaffold. Figure S3. Ebselen can covalently bind to ATG4B. (A) The purified recombinant ATG4B C74S mutant was subjected to SDS-PAGE. (B) Detailed characteristic peaks of ATG4B in denaturing mass spectrometry. Figure S4. Ebselen can promote ATG4B oligomerization to highly inhibit ATG4B. (A) Recombinant ATG4B (5 μM) was incubated with Ebselen-like analogues (50 μM) for 30 min and detected by non-reducing electrophoresis. (B) The purified recombinant ATG4B 2CS mutant was subjected to SDS-PAGE. (C) Recombinant ATG4B C74S mutant (5 μM) were treated with or without Ebselen (50 μM) and subjected to reducing or non-reducing electrophoresis. (D) A sketch of the redox properties of Ebselen. Ebselen cloud covalently bind to ATG4B Cys74 and induce oligomerization modification of ATG4B, which above regulated by the reducing agent DTT. [O] represents reactive oxygen species. Mono as monomer and Oligoes as oligomers. Figure S5. Ebselen suppresses the growth of CRC cells via ATG4B inhibition. (A) Western blot results of ATG4B expression in colonic epithelial cell line NCM460 and colon cancer cells. (B) WT and ATG4B KO HCT116 were treated with Staurosporine (STS, 2 μM) or Ebselen for 6 h and detected by western blot. (C) WT and ATG4B KO HCT116 [file 13578_2022_944_MOESM1_ESM.docx]
